# Solute Concentrations Influence Microbial Methanogenesis in Coal-bearing Strata of the Cherokee Basin, USA

**DOI:** 10.3389/fmicb.2015.01287

**Published:** 2015-11-18

**Authors:** Matthew F. Kirk, Brien H. Wilson, Kyle A. Marquart, Lydia H. Zeglin, David S. Vinson, Theodore M. Flynn

**Affiliations:** ^1^Department of Geology, Kansas State University ManhattanKS, USA; ^2^Division of Biology, Kansas State UniversityManhattan, KS, USA; ^3^Department of Geography and Earth Sciences, University of North Carolina at CharlotteCharlotte, NC, USA; ^4^Biosciences Division, Argonne National LaboratoryArgonne, IL, USA

**Keywords:** natural gas, unconventional reservoir, coal biodegradation, Cherokee basin, acetoclastic methanogenesis, hydrogenotrophic methanogenesis

## Abstract

Microorganisms have contributed significantly to subsurface energy resources by converting organic matter in hydrocarbon reservoirs into methane, the main component of natural gas. In this study, we consider environmental controls on microbial populations in coal-bearing strata of the Cherokee basin, an unconventional natural gas resource in southeast Kansas, USA. Pennsylvanian-age strata in the basin contain numerous thin (0.4–1.1 m) coalbeds with marginal thermal maturities (0.5–0.7% R_*o*_) that are interbedded with shale and sandstone. We collected gas, water, and microbe samples from 16 commercial coalbed methane wells for geochemical and microbiological analysis. The water samples were Na–Cl type with total dissolved solids (TDS) content ranging from 34.9 to 91.3 g L^−1^. Gas dryness values [C_1_/(C_2_ + C_3_)] averaged 2640 and carbon and hydrogen isotope ratios of methane differed from those of carbon dioxide and water, respectively, by an average of 65 and 183‰. These values are thought to be consistent with gas that formed primarily by hydrogenotrophic methanogenesis. Results from cultivation assays and taxonomic analysis of 16S rRNA genes agree with the geochemical results. Cultivable methanogens were present in every sample tested, methanogen sequences dominate the archaeal community in each sample (avg 91%), and few archaeal sequences (avg 4.2%) were classified within *Methanosarcinales*, an order of methanogens known to contain methylotrophic methanogens. Although hydrogenotrophs appear dominant, geochemical and microbial analyses both indicate that the proportion of methane generated by acetoclastic methanogens increases with the solute content of formation water, a trend that is contrary to existing conceptual models. Consistent with this trend, beta diversity analyses show that archaeal diversity significantly correlates with formation water solute content. In contrast, bacterial diversity more strongly correlates with location than solute content, possibly as a result of spatial variation in the thermal maturity of the coalbeds.

## Introduction

Microbial methanogenesis may be the future of energy extraction from subsurface hydrocarbon reservoirs (Meslé et al., 2013). Microbes can form methane, the primary component of natural gas, by degrading complex organic matter in coal, shale, and depleted oil reservoirs (Zengler et al., 1999; Jones et al., 2008). Moreover, communities of microorganisms capable of catalyzing these reactions are common in subsurface energy reservoirs (Strąpoć et al., 2011; Meslé et al., 2013; Head et al., 2014). By learning about the structure and function of these communities and environmental controls on their activity, we may be able to develop strategies to stimulate their growth and boost energy recovery (Gieg et al., 2008; Meslé et al., 2013; Hamilton et al., 2015; Ritter et al., 2015).

Microbial generation of methane from complex organic matter requires the combined effort of Bacteria and Archaea. Bacteria degrade complex organic matter and produce the relatively simple substrates that methanogenic Archaea use to make methane. Methanogenic substrates fall into three categories: (1) molecular hydrogen, formate, and certain alcohols, (2) methyl-containing C1 compounds such as methanol, methylamines, and methylsulfides, and (3) acetate (Whitman et al., 2006). Formation of methane from hydrogen is referred to as hydrogenotrophic methanogenesis whereas formation of methane from methyl-containing C1 compounds or the methyl group of acetate is referred to as methylotrophic methanogenesis (Costa and Leigh, 2014). Methylotrophs that use acetate can also be specifically referred to as acetoclastic methanogens.

Temperature is a primary control on methane formation in the subsurface. Microorganisms can tolerate a wide range of temperatures, but the upper temperature limit for microbial gas generation in subsurface hydrocarbon reservoirs appears to be around 80–90°C (Wilhelms et al., 2001). Where organic matter is exposed to temperatures above 70°C, natural gas can form instead by thermocatalytic reactions (Faiz and Hendry, 2006).

Other environmental controls on microbial conversion of organic compounds to methane include solute concentrations and nutrient availability (Head et al., 2014). Solute concentrations in subsurface hydrocarbon reservoirs range from levels consistent with freshwater to brine (Martini et al., 1998; Golding et al., 2013). Like temperature, microorganisms are capable of tolerating a wide range of salt concentrations (Pikuta et al., 2007; Waldron et al., 2007; Dong et al., 2014). However, the energy cost of adaptation fixes an upper salinity limit for growth and activity that may differ between groups of methanogens (Oren, 2011). Nutrient availability may affect how rapidly methane can form. In culturing experiments, addition of nutrients stimulates methane formation, suggesting that nutrient availability is one factor that could limit *in situ* rates (Harris et al., 2008; Gray et al., 2009; Jones et al., 2010; Glass and Orphan, 2012; Unal et al., 2012).

Coupled with these controls, properties of organic source materials themselves also influence microbial generation of natural gas. Biodegradation of solid- or liquid-phase hydrocarbons to simpler organic compounds and ultimately methanogenic substrates is initiated at the hydrocarbon-water interface. Hence, the surface area of that interface has the potential to influence how rapidly degradation can occur (Green et al., 2008; Wuchter et al., 2013; Head et al., 2014). The thermal maturity of coal influences the rate at which it can be converted to methane and the proportion of coal carbon that is convertible. As coal thermally matures, changes in composition decrease its bioavailability (Strąpoć et al., 2011).

Although we have learned much about microbial methanogenesis in subsurface hydrocarbon reservoirs, many questions remain unresolved. Little is known about microbial diversity in these systems, how geochemistry helps to shape those communities, impacts of commercial production activities (e.g., pumping history, hydraulic fracturing), controls on hydrocarbon bioavailability, steps in degradation pathways, and the timing of methanogenesis (Kirk et al., 2012; Golding et al., 2013; Wuchter et al., 2013; Gründger et al., 2015; Ritter et al., 2015).

In this study, we test the hypothesis that geochemistry has influenced microbial methane formation in coal-bearing strata of the Cherokee basin, an unconventional natural gas resource located in Kansas, USA. Our objectives are to (1) determine how natural gas in the strata formed, (2) assess whether cultivatable methanogens exist in formation water, (3) analyze microbial community composition, and (4) evaluate controls on community composition. To meet these objectives, we sampled water, gas, and microbes from 16 commercial coalbed methane wells. We analyzed water samples using chemical and isotopic techniques and microbial samples using sequencing and cultivation assays.

No previous studies have demonstrated how natural gas within Cherokee basin coal-bearing strata formed. However, gas isotope data primarily gathered from conventional wells completed in sandstone and carbonate reservoirs underlying the coalbeds suggest that a microbial gas component is present (Jenden et al., 1988; Lange, 2003). In addition to coal, potential energy sources for microbial methanogenesis in the strata include black shale and crude oil. The study area, therefore, allows us to examine subsurface microbial methanogenesis in the presence of diverse hydrocarbon compounds.

## Methods

### Field area

**Figure 1:**
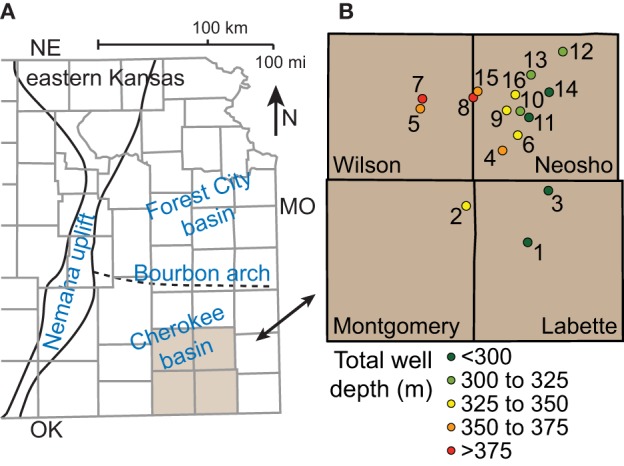
**Map showing (A) major structures in eastern Kansas and (B) locations and depths of coalbed methane wells sampled in the Cherokee basin**.

The Cherokee basin is located in southeast Kansas (Figure 1A) and extends for about 22,000 km^2^. It is bounded by the Ozark dome on its eastern margin, the Nemaha uplift to the west, the Arkoma basin to the south, and the weakly-defined Bourbon arch to the north (Lange, 2003). The sequence of Paleozoic bedrock in the Cherokee basin is similar to that in the Forest City basin, which lies just north of the Bourbon arch.

Coalbed methane has been produced from numerous middle and upper Pennsylvanian-age coalbeds in the Forest City and Cherokee basins. Over 25 coalbeds are recognized, however, most of the coalbeds exist within the Cherokee group of the Desmoinesian series (Bostic et al., 1993). The coalbeds are interbedded with layers of shale, sandstone, and limestone and dip gently (<0.5°) to the west across most of the basin. They are typically thin (0.4–1.1 m). As such, commercial gas wells in the Cherokee and Forest City basins are usually vertical and perforated at multiple coalbeds (Newell et al., 2012). Thin (0.6–1.5 m) black shales overlying some coalbeds likely also contribute gas to the wells (Newell et al., 2012). Lastly, the coalbeds are <760 m deep but were buried more deeply in the past. Since deposition of the Cherokee group, the area has been buried by about 1.5 km of late Pennsylvanian and Permian rocks, which have been subsequently eroded (Barker et al., 1992).

The thermal maturity of coal in the Cherokee basin approaches that needed to generate crude oil and natural gas via thermocatalytic reactions. Indeed small amounts of oil are produced from some Cherokee group wells (Newell et al., 1987). In terms of rank, a measure of thermal maturity, the coal is high-volatile bituminous A and B rank with vitrinite reflectance values commonly between 0.5 and 0.7% R_o_ (Jenden et al., 1988). Coal rank tends to increase with depth and distance south in eastern Kansas. These trends likely occur in response to burial heating combined with heating from warm, saline groundwater that entered the region from the south during the Ouachita orogeny (Bethke and Marshak, 1990; Wojcik et al., 1994).

Lange (2003) measured the gas content of coal and shale core and cuttings collected from the Cherokee group. The gas content of coal samples ranged widely, from 3 to over 300 standard cubic feet of gas per dry ton of coal (scf ton^−1^), whereas the gas content of black shale samples ranged from 3 to 35 scf ton^−1^.

### Commercial gas wells

With assistance from energy company personnel, we identified 16 commercial gas wells for sample collection (Table S1). The wells are located in Wilson, Neosho, Montgomery, and Labette counties (Figure 1B). The total depth of the wells increases with distance west across the field area, reflecting the dip of the strata, and ranges from 246 to 395 m. Each well is cased over its entire depth and perforated only over intervals where it passes through coalbeds. Specific intervals are recorded in 11 of the 16 well logs. For those wells, the sum of perforation interval length ranges from 2 to 8 m. The wells are between 8 and 12 years old. Gas production from the wells ranged between 17 and 64 mcf day^−1^ on average over the 2 weeks prior to sampling.

### Sample collection

We collected gas samples for chemical and isotopic analysis in Isotubes® (Isotech) at all 16 wells and water samples for chemical analysis from 15 wells. One well did not produce water during our visit. We collected unfiltered water samples for analysis of total alkalinity and filtered water samples for all other analytes: cations, anions, water isotopes, non-purgeable organic carbon (NPOC), and total nitrogen (TN). To filter samples, we used a peristaltic pump (Geotech) with Viton tubing and PFA filter housings to push water through a pre-combusted glass fiber membrane with 0.7 μm pores followed by a polyethersulfone membrane with 0.2 μm pores. We stored water samples in HDPE bottles and, in the case of NPOC and TN samples, pre-combusted amber glass bottles. To preserve cation, NPOC, and TN samples, we acidified them to pH <2 with concentrated HCl in the field.

We collected water samples for culturing from 13 wells in autoclaved (121°C for 30 min), nitrogen-flushed serum bottles. During sample collection, we inserted two hypodermic needles into the bottle, one to allow water to flow into the bottle directly from the well and another to vent displaced gas. We removed both needles simultaneously when the bottles were filled. By this approach, we avoided exposing the culture samples to oxygen.

We sampled microorganisms for sequencing by filtering formation water from 14 wells through mixed cellulose–ester filter membranes with 0.22 μm pores. Prior to sampling, we sterilized the membranes and filter housing by autoclaving at 121°C for 30 min. We pushed formation water through the filters using a sterile 60 mL syringe until the membrane became clogged with particulates, which may have included inorganic solids as well as biomass. The volume required to clog the filters ranged from 60 to 540 mL. Immediately after filtering, we preserved the samples with 0.2 mL of sucrose lysis buffer (Giovannoni et al., 1990) and plugged the filter housing with a luer lok plug. Although subsurface microbe samples collected by filtering water can be biased toward planktonic species (Flynn et al., 2008; Klein et al., 2008), water from coalbed methane wells typically contains coal fines that contribute attached species to a sample (Strąpoć et al., 2011).

We stored all of our samples on water ice in the field. In the laboratory, we stored water samples at 4°C and filtered microbe samples at −80°C.

### Geochemical analysis

We measured the temperature, pH, and electrical conductivity of water samples during sampling using an Oakton PC-300 meter. In the laboratory, we measured total alkalinity using Gran alkalinity titrations with 0.02 N H_2_SO_4_ titrant_._ We measured concentrations of cations (Na^+^, K^+^, Mg^2+^, Ca^2+^) and anions (Cl^−^, Br^−^, ${\text{SO}}_{4}^{2-}$) in water samples using ICS-1100 ion chromatographs (Thermo). The anion system was configured with carbonate eluent, an AS22 analytical column, and an AERS suppressor. The cation system was configured with sulfuric acid eluent, a CS12 analytical column, and a CERS suppressor. We analyzed sulfate concentrations of filtered, undiluted samples by ion chromatography after first removing chloride from the samples using OnGuard II Ag cartridges (Dionex). We measured total dissolved iron in filtered and acidified samples by first reducing the iron with hydroxylamine hydrochloride and then measuring ferrous iron concentration using the ferrozine method (Stookey, 1970) and a Genesys 10S UV–Vis spectrophotometer (Fisher). We measured NPOC and TN using Shimadzu TOC-L-CSH analyzer with a high salts kit.

We contracted Isotech Laboratories, Inc., to analyze the chemical and stable isotope composition of gas samples and the stable isotope composition of water samples. Gas composition was analyzed using gas chromatography. The carbon isotope composition of methane and carbon dioxide and the hydrogen isotope composition of methane were measured using dual-inlet isotope ratio mass spectrometry (DI-IRMS). Hydrogen isotopes were measured at 2‰ precision and carbon isotopes at 0.1‰ precision.

To test the significance of correlations in our chemical dataset, we used Spearman's Rho rank correlation tests. We carried out statistical calculations using Prism GraphPad software, version 6.00. We used two-tailed tests and considered *P* < 0.05 to be significant.

### Cultivation assays

We created five anaerobic cultures from each formation water sample. Three cultures tested the ability of cells to form methane from individual methanogenic substrates: acetate, methanol, and hydrogen. Two cultures were controls that constrained the initial methane content of the formation water and how much methane formed by consumption of compounds present in the raw formation water. We did not add substrates to either control and sterilized one of them by autoclaving (121°C, 30 min) following inoculation.

To assemble each culture, we injected 10 mL of formation water into a sterile, oxygen-free pressure tube containing a 1 mL amendment solution. The amendment solution contained macronutrients (50 μM ${\text{NH}}_{4}^{+}$ and 5 μM ${\text{PO}}_{4}^{3-}$; concentrations following dilution with sample), a reducing agent (100 μM Fe^2+^), and where appropriate, acetate (6.3 mM) or methanol (8.4 mM). We selected substrate concentrations based on their potential to generate methane. For each substrate, if microbes consumed the entire amount added, methane abundance in the culture headspace would rise to 9%. Initially the headspace gas was 95% nitrogen and 5% carbon dioxide in all of the cultures except those amended with hydrogen. In those cultures, the headspace gas was 55% nitrogen, 5% carbon dioxide, and 40% hydrogen.

The cultures incubated in the dark for 107 days at room temperature (~22°C). We then analyzed the methane content of each culture's headspace using a GOW MAC series 580 gas chromatograph equipped with a thermal conductivity detector.

### DNA extraction, amplification, and sequencing

We extracted total community DNA from the samples of filtered microorganisms using an UltraClean® Soil DNA Isolation Kit (MO BIO). We used the manufacturer recommended “Alternative Lysis Method” to limit DNA shearing. We measured DNA concentration and purity using a Nanodrop spectrophotometer (Fisher) and then contracted MR DNA® laboratory to amplify and sequence 16S rRNA genes in our samples.

The laboratory amplified DNA over 30 cycles of PCR using the HotStarTaq Plus Master Mix Kit (Qiagen) under the following conditions: 94°C for 3 min, followed by 28 cycles of 94°C for 30 s, and 53°C for 40 s and 72°C for 1 min. Following the final cycle, the reaction sequence included an elongation step at 72°C for 5 min. Reaction mixtures for amplification of Bacteria used primers 27F (AGRGTTTGATCMTGGCTCAG) and 519R (GTNTTACNGCGGCKGCTG) to cover variable regions V1 through V3 of the 16S rRNA gene. Reactions for Archaea used primers 349F (GYGCASCAGKCGMGAAW) and 806R (GGACTACVSGGGTATCTAAT) to cover variable regions V3 and V4.

After amplification, the laboratory verified amplification success using electrophoresis in 2% agarose gel. Multiple samples were then pooled together in equal proportions based on their molecular weight and DNA concentrations and purified using calibrated Ampure XP beads. The pooled and purified PCR product was used to prepare a DNA library by following Illumina TruSeq DNA library preparation protocol. Paired-end 2 × 250 sequencing was performed on an Illumina MiSeq system following manufacturer guidelines. Sequence data collected for this study are publically available over the internet through MG-RAST (Meyer et al., 2008) under project 15296.

### Analysis of sequencing data

The sequencing laboratory provided results in the form of fasta, quality, and mapping files. We processed sequencing data using QIIME v. 1.8.0 (Caporaso et al., 2010). We first split samples according to barcodes and filtered the sequences to remove low-quality reads (script: split_libraries.py). Next, we generated BIOM formatted OTU tables at 97% similarity and evaluated taxonomy with uclust (script: pick_de_novo_otus.py). The method used the Greengenes reference dataset (release 13_8; McDonald et al., 2012) and assigned the most detailed lineage description shared by at least 90% of the sequences within each OTU. Lastly, we removed singletons and created taxonomy tables (scripts: filter_otus_from_otu_table.py, summarize_taxa_through_plots.py).

We used QIIME to evaluate species richness for each sample (script: alpha_diversity.py, Chao1 method). To evaluate beta diversity, we first normalized the number of sequences in each sample to the number of sequences in the sample with the fewest, which was 68,841 for Archaea and 29,628 for Bacteria (script: single_rarefaction.py). Next, we exported Bray-Curtis dissimilatory matrices from QIIME (script: beta_diversity.py) and used them to generate non-metric multidimensional scaling (NMDS) ordination models in R (package: vegan, command: monoMDS). The analysis identified significant correlations between sample geochemistry and the NMDS models of relative abundance of individual phyla and sub-phyla (Bacteria) or orders (Archaea) using command envfit.

We used redundancy analysis (RDA) to evaluate the relative and combined influence of spatial distribution and geochemistry on archaeal and bacterial 16S rRNA gene community compositions (Borcard et al., 1992). We first applied Hellinger transformations to OTU distance values (package: vegan, command: decostand) and then evaluated the power and significance of the two datasets to explain variance in the transformed distance values using the rda command followed by the anova.cca command. Next, we selected reduced significant models (package: packfor, command: forward.sel) derived from the polynomial distribution of distances among samples (command: poly) and from the suite of geochemical variables (Blanchet et al., 2008). Lastly, we used the command varpart to partition the amount of variance explained by each model individually and combined.

## Results

### Geochemistry

**Figure 2:**
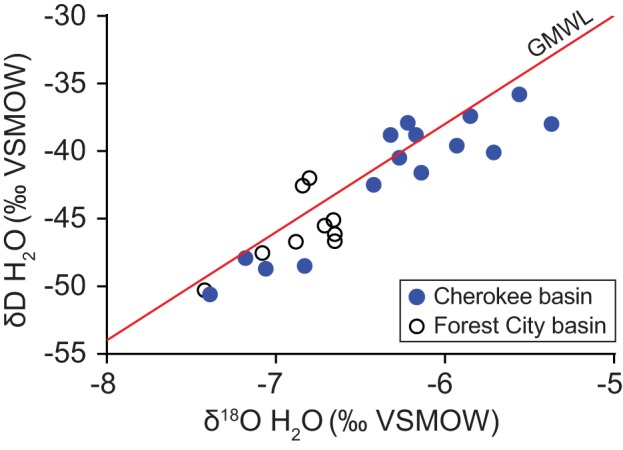
**Variation in the oxygen and hydrogen isotope ratios of water produced from coalbed methane wells in the Cherokee basin (this study) and the Forest City basin (McIntosh et al., 2008)**. Data are shown relative to the global meteoric water line (GMWL; Craig, 1961).

**Figure 3:**
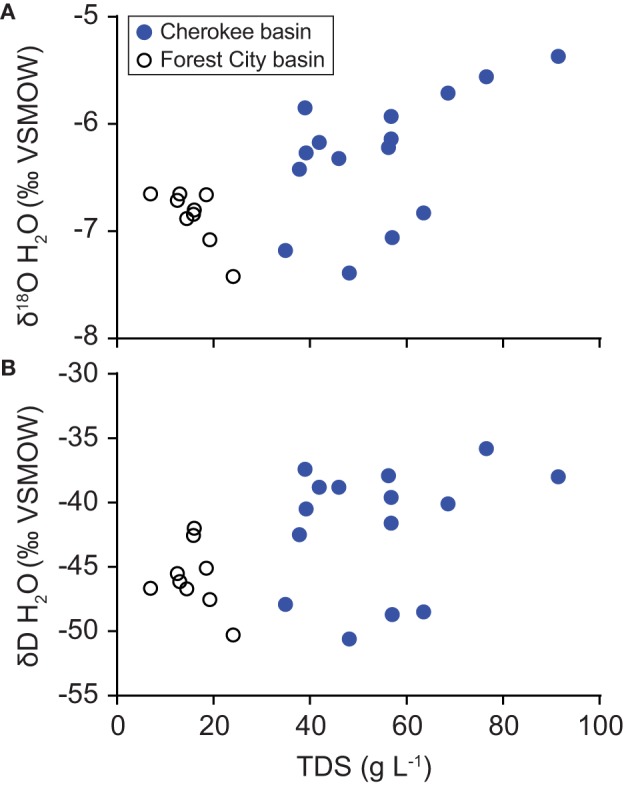
**Variation with TDS content in the oxygen (A) and hydrogen (B) isotope ratios of water samples collected from the Cherokee basin (this study) and the Forest City basin (McIntosh et al., 2008)**. For both basins, we calculated TDS-values using all available solute concentration data.

Table 1 summarizes geochemical results for water and gas. A detailed geochemistry dataset is available online (Tables S2, S3). Water samples were all sodium-chloride type. Total dissolved solids (TDS) concentrations, calculated based on measured ion concentrations, range from 34.9 to 91.3 g L^−1^. Water δ^18^O- and δD-values plot along the global meteoric water line (GMWL), near values observed by McIntosh et al. (2008) in samples from the Forest City basin (Figure 2). Water isotope ratios generally increase with TDS concentration (Figure 3). However, the correlations are not statistically significant.

**Table 1:** **Summary of geochemistry results**.

	**Min**	**Max**	**Avg**	***SD***
**PRODUCED WATER**				
pH	6.6	7.6	7.0	0.3
*T* (°C)	15.2	28.2	19.0	3.7
Cond. (mS cm^−1^)	46.4	79.6	60.9	10.2
Alk.(meq L^−1^)	3.3	8.5	4.9	1.5
Cl^−^ (M)	0.6	1.6	1.0	0.3
Br^−^ (mM)	0.9	3.7	1.8	0.8
${\text{SO}}_{4}^{2-}$ (μM)	7	112	50	22
Na^+^ (M)	0.5	1.2	0.8	0.2
K^+^ (mM)	1.7	4.7	2.6	0.9
Mg^2+^ (mM)	14.2	78.7	36.0	19.0
Ca^2+^ (mM)	11.7	58.5	31.8	12.5
Fe^2+^ (μM)	2	1502	372	438
NPOC (μM)	109	2018	295	483
TN (mM)	0.8	2.1	1.3	0.4
δD H_2_O^*^ (‰)	−50.6	−35.8	−41.8	4.8
δ^18^O H_2_O (‰)	−7.4	−5.4	−6.3	0.6
**GAS**				
CH_4_ (mol %)	95.3	98.5	97.3	0.1
CO_2_ (mol %)	0.5	1.7	1.0	0.4
C_2_H_6_ (mol %)	0.02	0.27	0.06	0.07
C_3_H_8_ (mol %)	0.0003	0.23	0.02	0.06
δD CH_4_ (‰)	−228.2	−217.2	−222.2	2.9
δ^13^C CH_4_ (‰)	−70.0	−56.5	−60.9	4.0
δ^13^C CO_2_ (‰)	−5.4	9.2	4.3	3.9

* *Oxygen and hydrogen delta values are relative to VSMOW. Carbon delta values are relative to VPDB*.

**Figure 4:**
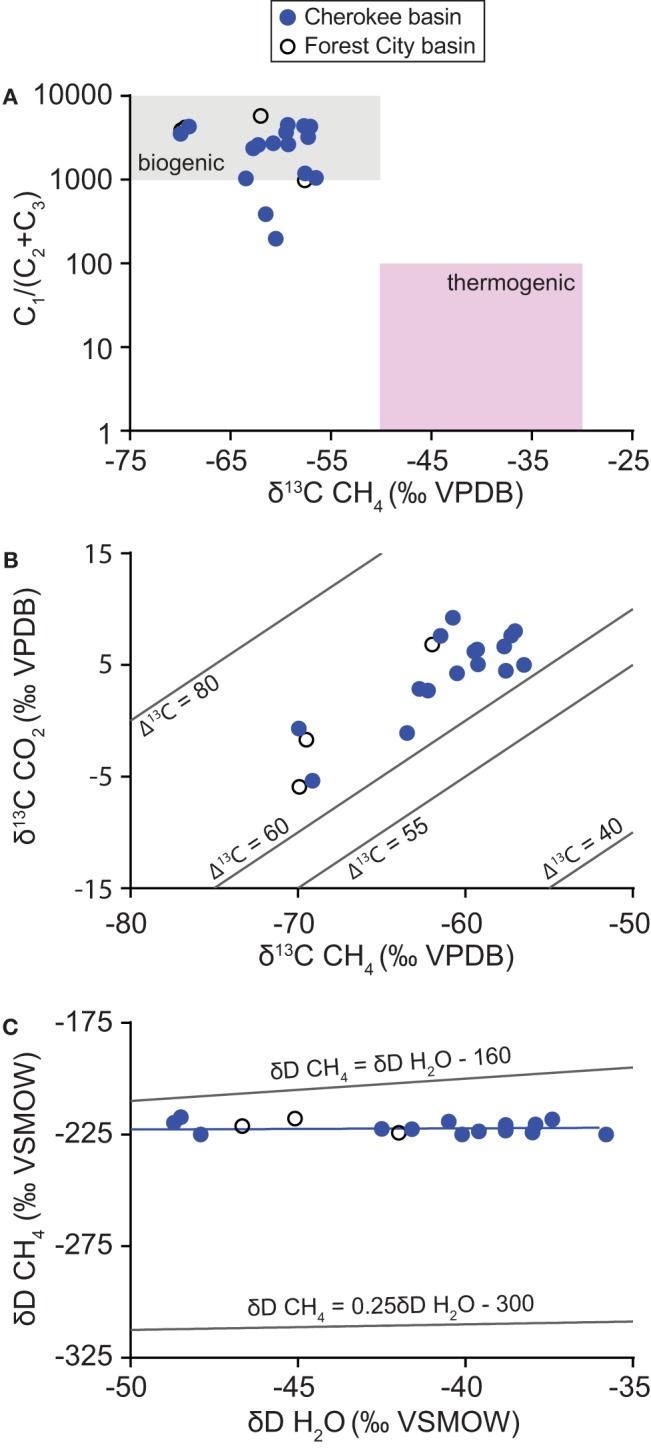
**Variation in (A) gas dryness index values [C_1_/(C_2_+ C_3_)] relative to the carbon isotope ratio of methane, (B) carbon isotope ratios of carbon dioxide relative to methane, and (C) hydrogen isotope ratios of methane relative to water**. Data were collected from coalbed methane wells in the Cherokee basin (this study) and the Forest City basin (McIntosh et al., 2008). Δ^13^C-values in **(B)** are calculated as difference in carbon isotope ratios of carbon dioxide and methane (Δ^13^C = δ^13^C CO_2_− δ^13^C CH_4_). Graph **(C)** includes a best-fit line through Cherokee basin data (δD CH_4_ = 0.05δD H_2_O − 220) and lines defined by Whiticar (1999) for hydrogenotrophic and methylotrophic methanogenesis (Equations 1 and 2, respectively).

Gas samples were composed almost entirely of methane with an average gas dryness index [C1/(C2 + C3)] of 2640 (Figure 4). Methane δ^13^C-values differ from the δ^13^C-values of carbon dioxide by an average of 65‰ (Δ^13^C = δ^13^C CO_2_− δ^13^C CH_4_). Methane δD-values differ from the δD-values of corresponding water samples by an average of 183‰ (ΔD = δD H_2_O − δD CH_4_).

**Figure 5:**
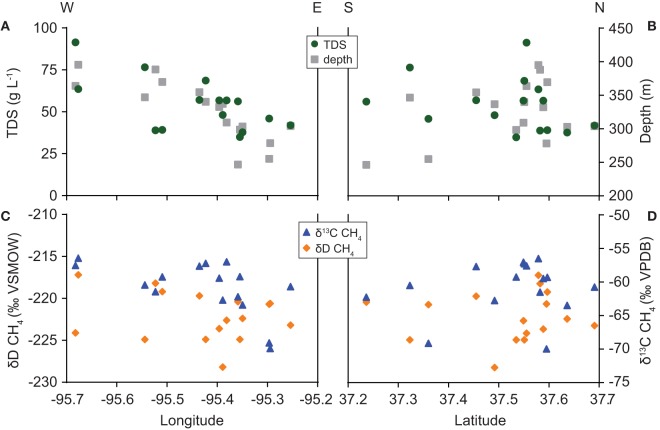
**Spatial variation in (A,B) TDS concentration and total well depth and (C,D) carbon and hydrogen isotope ratios of methane**.

The composition of water and gas samples varied with well location (Figure 5). TDS concentration increases with distance west across the field area, paralleling the trend in total well depth. The correlation between longitude and TDS concentration is significant (ρ = −0.61, *P* = 0.017). However, solute levels show no significant north-south trend. Similarly, methane δ^13^C-values share a significant correlation with longitude (ρ = −0.60, *P* = 0.016) but not latitude. Methane δD-values, as well as water δD and δ^18^O-values (not shown), do not correlate significantly with latitude or longitude in our dataset.

### Cultivation assays

**Figure 6:**
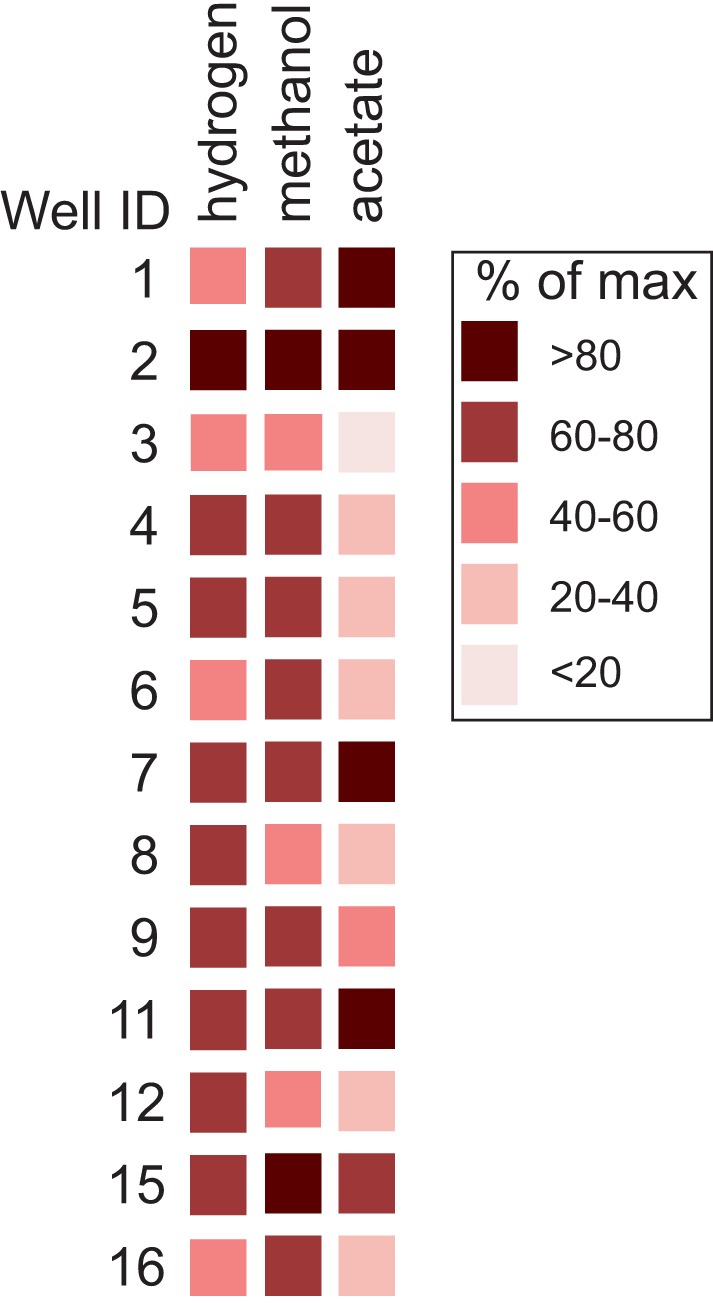
**Abundance of methane that formed in cultures relative to the maximum amount possible per substrate**. Results are normalized to levels of methane observed in control cultures.

Methane levels increased above control levels in all of the cultures amended with substrate (Figure 6; Table S4). The amount of methane that formed in cultures amended with hydrogen and methanol was nearly uniform. Headspace methane abundance increased to an average of 67% (*SD* = 13) and 68% (*SD* = 10) of the maximum amount possible in the hydrogen and methanol cultures, respectively. In contrast, methanogenesis from acetate was more variable. The amount that formed in cultures amended with acetate averaged 53% (*SD* = 34) of the maximum amount possible.

### Alpha diversity and taxonomy

DNA concentrations in our extracted solutions equate to 476 to 8640 ng of DNA per liter of formation water (Table S5). 16S rRNA genes were successfully amplified and sequenced in nine samples for Archaea and twelve for Bacteria. After processing the raw sequencing data, the number of archaeal and bacterial sequences from each sample averaged 181,245 and 60,997, respectively. From those sequences, the total number of OTUs defined at 97% similarity is 3774 and 8777, respectively, in the archaeal and bacterial datasets. Reflecting these differences, Chao1-values were smaller for the archaeal (avg 1647) dataset than the bacterial dataset (avg 2189).

**Figure 7:**
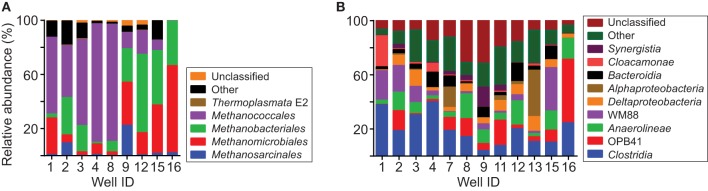
**Taxonomic distribution of 16S rRNA sequences**. Chart **(A)** shows the distribution of sequences classified within orders of Archaea. Relative abundances are shown for all of the groups of methanogens detected in the analysis. Chart **(B)** shows the distribution of sequences grouping within classes of Bacteria. For clarity, only classes with average relative abundance per sample >2% are specifically identified.

On average, 91% of the archaeal sequences were classified within orders of methanogens (Figure 7A, Table S6). Sequences classified within *Methanococcales* had the highest relative abundance (avg 41.2%). Sequences classified within *Methanomicrobiales* and *Methanobacteriales* were also generally abundant (avg 21.5 and 23.9%, respectively), whereas those classified within *Methanosarcinales* and *Thermoplasmata* order E2 were generally low in relative abundance (avg 4.2 and 0.2%, respectively). In addition to these groups, an average of 1.7 and 7.4% of archaeal sequences were not classified or classified in other groups of Archaea, respectively.

Most of the bacterial sequences were classified within the following classes (avg relative abundance per sample): *Clostridia* (19.5%), *Actinobacteria* class OPB41 (10.5%), *Anaerolineae* (9.6%), WM88 of phylum Hyd24-12 (8.6%), *Deltaproteobacteria* (5.8%), Bacteroidia (5.6%), *Alphaproteobacteria* (4.9%), *Cloacamonae* of phylum WWE1 (3.1%), and *Synergistia* (2.5%) (Figure 7B, Table S7). In addition to these groups, 16% of the sequences were classified within classes with an average relative abundance <2 and 14% of the sequences were unclassified.

### Beta diversity

**Figure 8:**
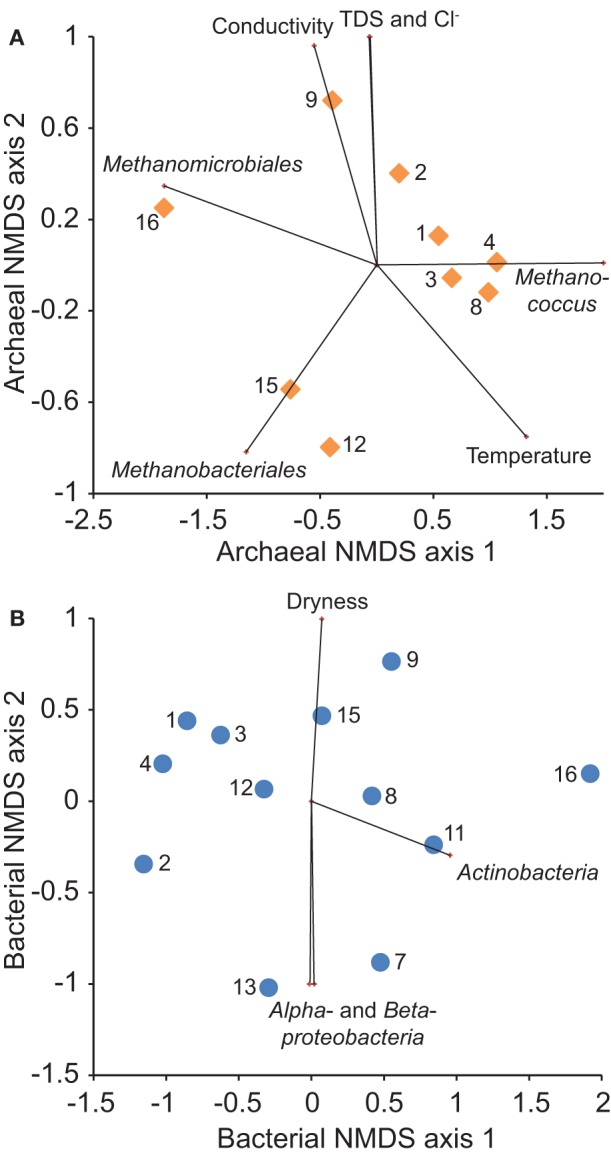
**Non-metric multidimensional scaling (NMDS) ordination of the Bray-Curtis similarity of relative abundance of (A) archaeal and (B) bacterial 16S rRNA gene OTUs among samples (numbered symbols)**. Overlying vectors show significant (*P* < 0.05) correlations of microbial community composition with geochemistry and major taxonomic group abundance.

The best NMDS models for both archaeal and bacterial 16S rRNA gene sequences were two-dimensional (stress = 0.0391, 0.127, respectively). Each model correlated with multiple geochemical variables and relative population sizes (Figure 8). Archaeal heterogeneity relates significantly (*P* < 0.05) to differences in the relative abundance of sequences classified in *Methanomicrobiales, Methanobacteriales*, and *Methanococcus*, and was correlated significantly (*P* < 0.05) with conductivity, TDS content, chloride concentration, and temperature. Bacterial heterogeneity relates significantly (*P* < 0.05) to differences in the relative abundance of sequences classified in *Actinobacteria, Alphaproteobacteria*, and *Betaproteobacteria*, and was correlated weakly (*P* = 0.064) with gas dryness index values.

Redundancy analysis indicates that bacterial community composition varies significantly with location whereas archaeal community composition varies significantly with geochemistry (Table 2). Including spatial variables in a combined model, however, does improve model fit for Archaea. The spatial effect on microbial community composition may explain separation of sample 16 from samples 1–4 along NMDS1 axes for both models (Figure 8). Variation along NMDS 2 axes appears to be related to factors other than location.

**Table 2:** **Influence of spatial and geochemical factors on microbial community composition**.

	**RDA-test**		**Reduced RDA**	
	***r*^2^**	***P***	***r*^2^**	***P***
**ARCHAEA**				
Location	0.215	0.16	0.103	0.16
Geochemistry^*^	0.654	0.04	0.54	0.02
Location + geochemistry^*^	–	–	0.603	0.02
**BACTERIA**				
Location^*^	0.149	0.03	0.064	0.03
Geochemistry	0.226	0.18	0.054	0.1
Location + geochemistry	–	–	0.113	0.1

* *Indicates statistically significant relationship (P < 0.05)*.

## Discussion

### Gas origin

Our geochemical results indicate that the gas we sampled was generated primarily by microbial reactions. Geochemical tracers for natural gas origin include gas dryness index as well as carbon and hydrogen isotopic compositions (Golding et al., 2013). Compared to gas generated by thermocatalytic reactions, natural gas formed by microbial reactions tends to have a higher dryness index and contain methane with a more negative δ^13^C (Bernard et al., 1978). Dryness and δ^13^C-values for our samples fall near or within limits consistent with a microbial origin (Figure 4A). If some thermogenic component is present, then it is minor based on the compositions we observe.

Analysis of the microbial community supports this interpretation. Cultivable methanogens were present in every water sample. Relative abundances of archaeal 16S rRNA sequences indicate that the archaeal community is dominated by methanogens (Figure 7A). Moreover, Bacteria needed to supply methanogenic substrates are also present (Figure 7B). Members of bacterial class *Clostridia* have been detected in many other subsurface energy resources and appear to play a major role in hydrocarbon biodegradation (Strąpoć et al., 2011; Meslé et al., 2013; Head et al., 2014). Genomic evidence indicates that members of class *Anaerolinaea*, as well as other *Chloroflexi*, degrade complex organic matter and contribute significantly to subsurface carbon cycling (Hug et al., 2013). Microorganisms in uncultured bacterial phylum WWE1 have been implicated in cellulose degradation in anaerobic sludge digestors (Limam et al., 2014). Overall, these results show that microbial constituents needed to degrade hydrocarbons and generate natural gas occur in Cherokee basin coal-bearing strata.

### Contribution of hydrogenotrophic methanogens

Our analysis of the archaeal community suggests that methane formed primarily by hydrogenotrophic methanogenesis. The ability to use hydrogen as a substrate is widespread among methanogens (Whitman et al., 2006; Thauer et al., 2008). In contrast, methanogens that can use acetate and methyl-containing C1 compounds exist primarily in order *Methanosarcinales* (Thauer et al., 2008). The low relative abundance of sequences classified within *Methanosarcinales* on average, therefore, suggests that hydrogenotrophs played a dominant role overall in generating the gas we sampled.

Carbon isotopes of methane and carbon dioxide support this interpretation. Methane δ^13^C-values commonly differ from the δ^13^C of associated carbon dioxide by 60 to 80‰ where gas is formed by hydrogenotrophic methanogens and <55‰ where gas is formed by methylotrophs (Jenden and Kaplan, 1986; Whiticar et al., 1986; Whiticar, 1999; Golding et al., 2013). Differences in δ^13^C-values for methane and carbon dioxide that we observed (Figure 4B), therefore, are consistent with methane generated primarily by hydrogenotrophic methanogenesis.

Hydrogen isotopes of methane and water provide a third line of evidence in support of our interpretation. Where hydrogenotrophs generate methane, δD-values of methane and water are typically related to one another according to (Whiticar, 1999):

(1) $$\delta \text{D }{\text{CH}}_{4}=\delta \text{D }{\text{H}}_{2}\text{O }-\text{ 16}0(\pm \text{1}0)\%$$

By comparison, where methylotrophic methanogens generate methane, the offset between δD ratios of methane and water are much greater (Whiticar, 1999):

(2) $$\delta \text{D }{\text{CH}}_{4}=\;0.\text{25}\delta \text{D }{\text{H}}_{2}\text{O}-\beta$$

where β is thought to range between 300 and 377‰. Hydrogen isotope ratios for our samples plot closest to the line thought to represent hydrogenotrophic methanogenesis (Figure 4C). Therefore, our hydrogen isotope data are consistent with hydrogenotrophic methanogenesis as the primary source of methane in our samples.

A dominance of hydrogenotrophic methanogens is not uncommon in coalbed methane reservoirs. Coalbeds found to be dominated by hydrogenotrophs based on archaeal community analysis include those in the Illinois basin (Strąpoc et al., 2008), Ishikari basin (Shimizu et al., 2007), Jharia basin (Singh et al., 2012), and Qinshui basin (Guo et al., 2014).

### Contribution of methylotrophic methanogens

**Figure 9:**
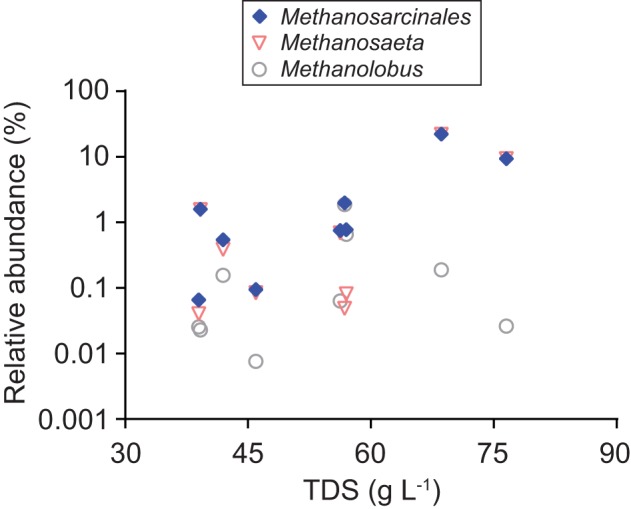
**Variation with TDS content in the relative abundance of archaeal 16S rRNA sequences classified within the order ***Methanosarcinales*** and genera ***Methanosaeta*** and ***Methanolobus*****.

Although hydrogenotrophs appear to have been dominant overall, our results indicate that methylotrophic methanogens were also involved and that their contribution increased with the solute content of formation water. The relative abundance of sequences classified within *Methanosarcinales* is low on average but ranged up to 22% in our samples (Figure 7). Relative abundance values share a significant positive correlation with the TDS content of formation water (ρ = 0.77, *P* = 0.02; Figure 9).

Sequences classified within *Methanosarcinales* were nearly all further classified within two genera: *Methanosaeta* and *Methanolobus* (Figure 9). Of the two, sequences classified within *Methanosaeta* were by far more abundant, accounting for 92% of the *Methanosarcinales* sequences overall. Members of genus *Methanosaeta* require acetate as a substrate for methanogenesis (Kendall and Boone, 2006). The results indicate, therefore, that the primary pathway of methylotrophy in the coalbeds is acetoclastic methanogenesis. As such, the increase in the relative contribution of methylotrophy with formation water salinity appears to be driven primarily by an increase in the contribution of acetoclastic methanogenesis.

**Figure 10:**
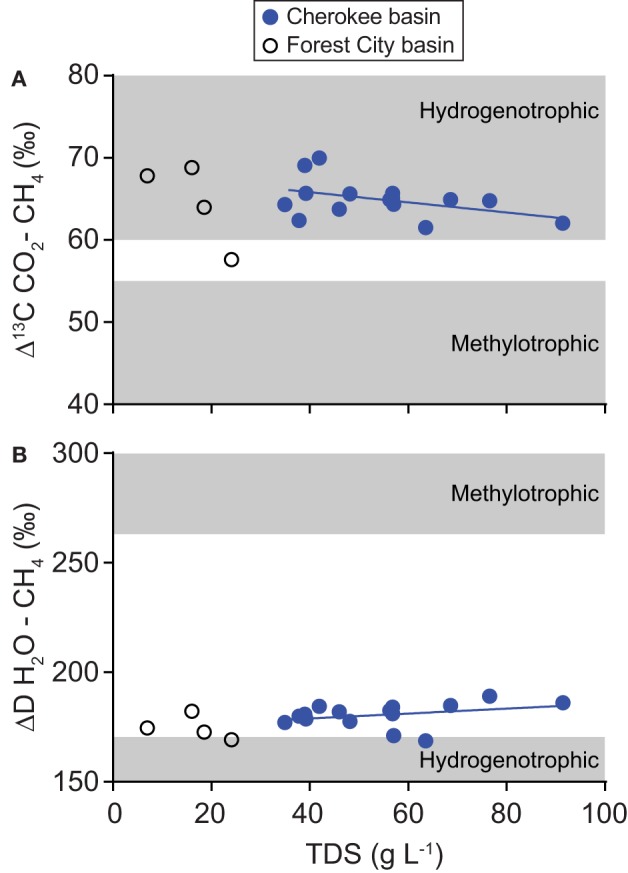
**Variation relative to TDS content in (A) differences between carbon dioxide and methane carbon isotope ratios (Δ^13^C) and (B) differences between water and methane hydrogen isotope ratios (ΔD)**. Data were collected from coalbed methane wells in the Cherokee basin (this study) and the Forest City basin (McIntosh et al., 2008). For both basins, we calculated TDS-values using all available solute concentration data. Best-fit lines through Cherokee basin data are shown on each graph.

Results of our isotopic analyses support this interpretation. Isotopic tracers of gas origin trend toward values indicative of methylotrophic methanogenesis as the TDS content of formation water increases (Figure 10). Including data from Forest City basin coalbed methane wells allows us to examine these trends over a broader range of solute concentrations than possible within our dataset alone. In the combined dataset, the correlation of TDS with Δ13C CO_2_–CH_4_ is not statistically significant (ρ = −0.24, *P* = 0.333) but the correlation with ΔD H_2_O–CH_4_ is significant (ρ = 0.47, *P* = 0.045).

Compared to hydrogenotrophic and acetoclastic methanogenesis, less is known about isotope fractionation during methanogenesis from methyl-containing C1 compounds. Researchers have concluded that stable isotopes cannot be used to distinguish methane formed from methyl-containing C1 compounds from methane formed by acetoclastic methanogenesis (e.g., Strąpoć et al., 2011; Golding et al., 2013). However, laboratory studies have reported large carbon isotope fractionations during methanogenesis from methyl-containing C1 compounds, similar to those observed during hydrogenotrophic methanogenesis (Krzycki et al., 1987; Conrad and Claus, 2005; Penger et al., 2012). Nonetheless, this source of uncertainty does not affect our interpretation because our analysis of the archaeal community indicates that methanogenesis from methyl-containing C1 compounds was minimal.

We did not observe any significant relationship between the amount of methane that formed from acetate in cultures and formation water salinity or any other variable. The absence of a significant relationship may reflect culturing under ideal conditions. Substrates and essential nutrients are rarely found in abundance *in situ*. Differences in conditions between the cultures and coalbeds likely caused differences in the identity and activity of microbes in each setting. Secondly, our cultures were all amended with an equal amount of acetate. In contrast, the flux of acetate generated by bacteria in the coalbeds may vary spatially and temporally. Such variation may contribute to variation in the proportion of methane formed by acetoclastic methanogens. Lastly, acetoclastic methanogen growth may have been slow compared to methanogens using hydrogen and methanol, delaying the complete conversion of acetate to methane in some of the cultures (e.g., Alperin et al., 1992).

### Comparison to existing conceptual models

An increase in the proportion of acetoclastic methanogenesis with salinity is contrary to existing conceptual models that relate salinity to metabolic pathways of methanogenesis. Traditionally, acetoclastic methanogenesis is thought to be the primary metabolic pathway in freshwater environments whereas hydrogenotrophic methanogenesis is the major pathway in saline systems (Whiticar et al., 1986; Whiticar, 1999; Hornibrook et al., 2000). Although the processes could coexist in each setting, this conceptual model does not predict that the significance of acetoclastic methanogenesis would increase with salinity.

Consistent with the traditional model, Oren (2011) describes a bioenergetic basis for shifts in substrate use with solute levels. As noted in the Introduction Section, adaptation to elevated solute concentration consumes energy (Oren, 2011). As a result, the upper salinity concentration limit for a microorganism varies with the energy yield of its metabolic reaction. Typically methyl-containing C1 compounds are the most energetically favorable methanogenic substrates and acetate the least (Head et al., 2014). These relationships imply that the upper salinity limit is highest for methanogenesis from methyl-containing C1 compounds and lowest for methanogenesis from acetate (Oren, 2011).

Why our results differ from the traditional conceptual model is unclear. One possibility is that the energetics of methanogenic reactions in our system do not follow the assumed order. For example, acetoclastic methanogenesis may be more energetically favorable than hydrogenotrophic methanogenesis in zones with elevated solute levels in our system. To test this hypothesis, additional data are needed to fully constrain the bioenergetics of each microbial reaction *in situ*. In addition, differences between our results and the traditional model may occur in response to controls other than energetics. The distribution of microbially-catalyzed reactions does not always follow a strict thermodynamic hierarchy because physiological, ecological, and kinetic factors also influence reaction rates (e.g., Bethke et al., 2011; Hansel et al., 2015).

### Controls on microbial community composition

Beta diversity analysis results for Archaea examine environmental controls on the archaeal community within a broader context than our discussion of metabolic pathways above. Nonetheless, the results are consistent. Of the variables we tested, the composition of the archaeal community is most closely related to variation in formation water salinity. Improvement of model fit by considering location as well as geochemical variables may occur because formation water solute levels vary spatially (Figure 5). Alternatively, this relationship may also reflect spatial variation in the bacterial community.

The significant relationship between bacterial community and location may reflect variation in coal thermal maturity. As coal thermally matures, its chemical composition, structure, and bioavailability evolves (Strąpoć et al., 2011). We hypothesize that such changes would affect which enzymes and other biological macromolecules are needed for degradation and thus the composition of the microbial community that is able to catalyze the reactions. Spatial variability in formation water geochemistry cannot be ruled out as a possible mechanism for the correlation between location and bacterial community composition. However, the lack of a significant correlation with geochemistry directly suggests otherwise.

### Uncertainty in sequencing data

Our microbial community analysis relies on variation in relative abundance of 16S rRNA genes. Potential sources of error in our analysis include bias introduced by primer specificity and the sequencing platform. A recent analysis of bias associated with MiSeq indicates that errors introduced by the sequencing platform have a relatively minor effect on quantitative abundance estimations compared to primer choice (Tremblay et al., 2015). For the primer combinations used in our study, theoretical coverages calculated with the Ribosomal Database Project Probe Match algorithm are 68 and 81% for the primers used for Bacteria and Archaea, respectively, with no obvious systematic bias. While no primer set is universal, comparative analysis among samples with matched data types are largely robust if protocols are applied consistently (Tremblay et al., 2015), as was the case for our study.

### Implications

Our findings imply that microbial activity has contributed significantly to energy resources across eastern Kansas. Based on geochemical data and cultivation assays, McIntosh et al. (2008) interpreted that natural gas in Forest City basin coalbeds also formed via microbial reactions. Combining their results with our own, therefore, indicates that middle and upper Pennsylvanian coalbeds across eastern Kansas are biogenic natural gas reservoirs.

Secondly, our results imply that acetoclastic methanogens may be more important in high salinity environments than previously considered. The upper salinity limit for acetoclastic methanogenesis is not well-known (Oren, 2011). Previous studies estimated the limit to be near 60 g L^−1^ (Oren, 2002; Waldron et al., 2007). Oremland and Miller (1993) report evidence for acetoclastic methanogenesis in incubations of sediment from an alkaline (pH 9.7), highly-saline lake (up to 141 g L^−1^). However, the salinity of water that was present in their experiments is unclear. Our cultivation assays, stable isotope analyses, and analysis of the archaeal community provide compelling evidence that acetoclastic methanogens contributed significantly to methane formation in water with as much as 91 g L^−1^ TDS content. Stable isotope studies may overlook the contribution of acetoclastic methanogenesis in saline environments if they assume results consistent with methylotrophic methane could have only come from consumption of methyl-containing C1 compounds.

Lastly, our findings imply that human activities have the potential to influence the pathway of methanogenesis in subsurface hydrocarbon reservoirs by affecting the solute content of formation water. Potential human activities that could alter formation water solute levels include injection of fluids for hydraulic fracturing and enhanced/tertiary oil recovery (e.g., Kirk et al., 2012; Wuchter et al., 2013; Cluff et al., 2014). Pumping to extract natural gas also has the potential to alter water chemistry by drawing water into a hydrocarbon reservoir from an adjacent formation (Kirk et al., 2012).

## Conclusions

In this study we consider microbial methanogenesis in Cherokee basin coal-bearing strata. Using microbial analyses and geochemical tracers of gas origin, we shed light on the origin of gas in the coalbeds and environmental controls on microbial community composition. Our results suggest that natural gas in the strata is biogenic and that microorganisms needed to form methane persist. Hydrogenotrophic methanogens appear to have been primarily responsible for generating the methane we sampled. However, acetoclastic methanogens also contributed and the proportion of gas they generated appears to increase with the solute content of formation water. This trend is contrary to existing conceptual models that predict that acetoclastic methanogenesis would increase in relative significance as salinity decreases. Consistent with our interpretation, archaeal diversity is more strongly correlated to variation in formation water salinity than any other variable we examined. Location is also relevant, however, possibly as a result of spatial variation in solute levels and bacterial community composition. In contrast to Archaea, bacterial diversity more strongly correlates with location than salinity, possibly as a result of spatial variation in the thermal maturity of the coalbeds.

## Author contributions

MK designed the project. MK, BW, KM, and DV performed field work. Each author participated in analysis of samples and data. MK wrote the manuscript, with significant input from each co-author.

### Conflict of interest statement

The authors declare that the research was conducted in the absence of any commercial or financial relationships that could be construed as a potential conflict of interest.
